# Development of bullous lung disease with pneumothorax following SARS‐CoV‐2 infection

**DOI:** 10.1002/rcr2.1013

**Published:** 2022-08-02

**Authors:** Hafizah Abdullah, Yen Shen Wong, Muhammad Amin Ibrahim, Aisya Natasya Musa, Thevaraajan Jayaraman, Mohd Arif Mohd Zim

**Affiliations:** ^1^ National Heart Institute (IJN) Kuala Lumpur Malaysia; ^2^ Faculty Of Medicine University Teknologi MARA (UiTM) Sg Buloh Selangor Malaysia

**Keywords:** bullous lung disease, pneumothorax, severe acute respiratory syndrome coronavirus 2 (SARS‐CoV‐2) infection

## Abstract

Cystic lung formation secondary to severe acute respiratory syndrome coronavirus 2 (SARS‐CoV‐2) infection was described during coronavirus disease pandemic, but with relatively low prevalence. A rare yet under‐recognized complication is that these cystic areas may progress to bullae, cavities and pneumothorax. We reported two cases of ruptured bullae with pneumothorax following SARS‐CoV‐2 infection. Two patients were discharged following SARS‐CoV‐2 pneumonia, which did not require invasive mechanical ventilation (IMV). However, both patients presented again a month later with shortness of breath. Repeated computed tomography (CT) thorax showed development of bullous lung disease and pneumothorax. The first patient underwent surgical intervention whilst the second patient was treated conservatively. Development of bullous lung disease following SARS‐CoV‐2 infection is rare but may be associated with serious morbidity. Patients whose general condition permits should be offered surgical intervention whilst conservative management is reserved for non‐surgical candidates.

## Graphical abstract

Cystic lung formation secondary to COVID‐19 infection was described during COVID‐19 pandemic, but with relatively low prevalence. We described two patients who develop bullous lung disease with pneumothorax post COVID‐19 infection.

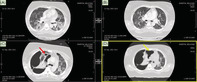

## INTRODUCTION

Cystic lung formation secondary to severe acute respiratory syndrome coronavirus 2 (SARS‐CoV‐2) infection was described during the coronavirus disease pandemic, but with relatively low prevalence. A rare yet under‐recognized complication is that these cystic areas may progress to bullae and cavity formation, thereby predispose to spontaneous pneumothorax. The significance of pneumothorax in SARS‐CoV‐2 infection was initially limited to several case reports/series, however it has been increasingly described and analysed in multiple observational studies during the ongoing pandemic.^1^, ^2^ We reported two cases of ruptured bullae with pneumothorax who were readmitted 1 month following SARS‐CoV‐2 infection. Both patients have no pre‐existing lung disease prior SARS‐CoV‐2 infection.

## CASE REPORT

### Case 1

**FIGURE 1:**
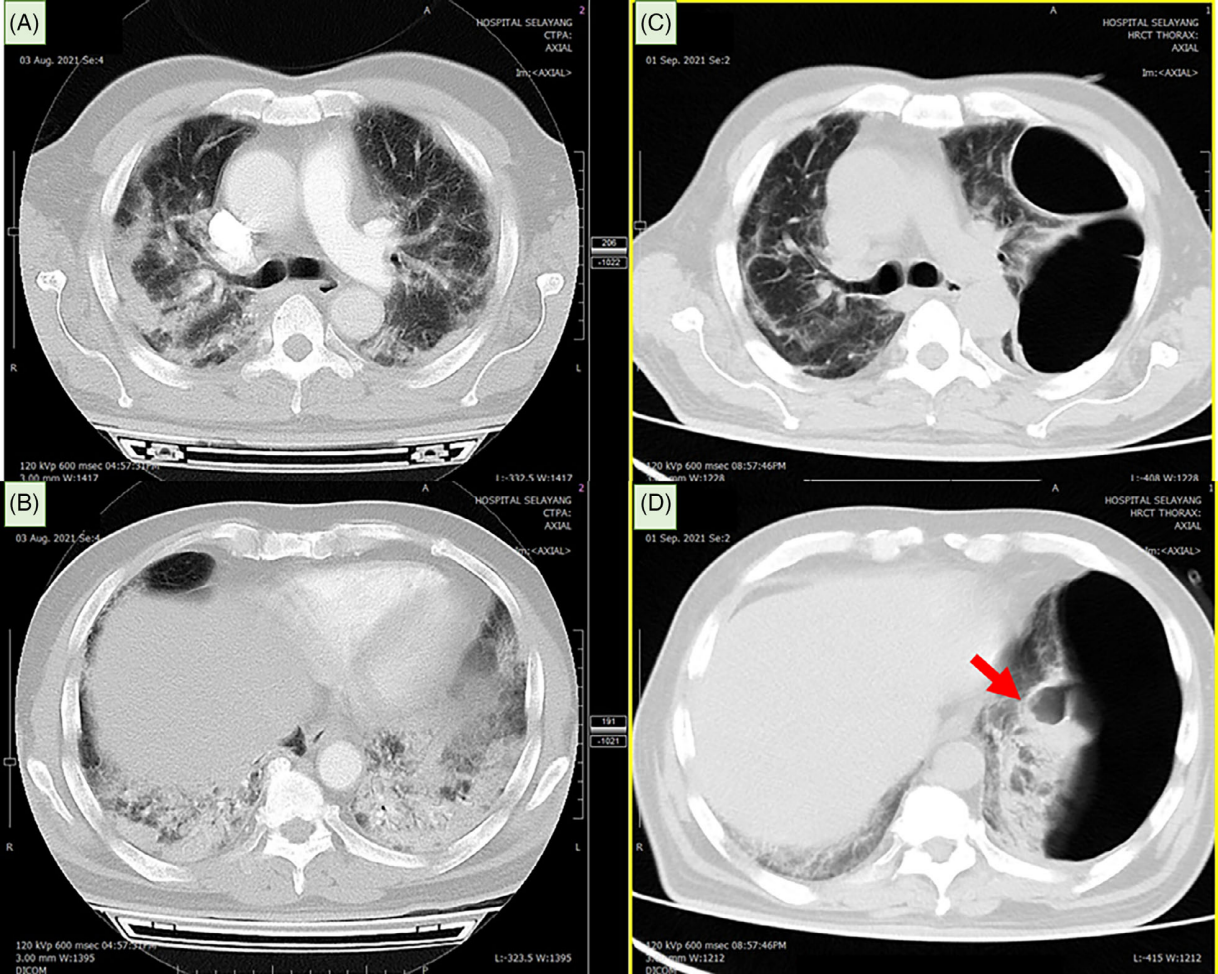
CT thorax at day 4 of SARS‐CoV‐2 infection (image A and B) revealed patchy consolidation in both lungs, predominantly at bilateral peripheral and lower lobe distribution. A repeat CT thorax at 1 month post SARS‐CoV‐2 infection (image C and D) showed bullous development with ruptured left lower lobe bullae leading to loculated pneumothorax (red arrow). The compressive effect of pneumothorax contributed to left lower lobe collapse and mediastinal shift to the right.

A 60‐year‐old man presented with worsening breathlessness and left‐sided pleuritic chest pain over 2 days. He had a background history of Diabetes Mellitus on single oral hypoglycemic agent with latest HbA1c of 9.2%. He was discharged a month previously following SARS‐CoV‐2 pneumonia with pulmonary embolism in the segmental branches of right pulmonary artery (Figure 1A,B). He required face mask oxygen at 5 L/min upon initial presentation and was treated with dexamethasone, baricitinib, and anticoagulant as per local guidelines. He was discharged well with tapering dose of prednisolone and novel oral anticoagulant after 25 days of hospitalization.

On recent presentation, he was afebrile, tachypneic with respiratory rate 24 breaths/min, tachycardic with heart rate 119 beats/min and oxygen saturation of 91% on 2 L/min nasal cannula. Clinical examination revealed reduced breath sounds and left lower zone fine crepitations. Computed tomography (CT) thorax showed a large bulla noted occupying almost the entire left mid to lower hemithorax (measuring 15.1 × 8.3 × 15.2 cm) with minimal septation noted within the superior and inferior part of this bulla. In addition, the present bulla caused a compressive effect to the left lung causing near total collapse of the left lower lobe with mediastinal shift to the right (Figure 1C,D). The findings were absent in the recent imaging a month ago.

A chest tube was inserted and subsequently it was complicated with pseudomonas empyema which required posterior left thoracotomy with decortication and lung parenchyma repair. Intraoperative findings noted an air leak over the lower segment of the left upper lobe, with minimal pus and thickened cortex. The air leak was repaired using prolene 4/0 pledgetted, followed by adhesionolysis and decortication. Post‐operatively, he had a good recovery and serial imaging showed resolution of pneumothorax. He was discharged after 42 days of inpatient pulmonary rehabilitation.

### Case 2

A 38‐year‐old woman presented with worsening dyspnea and pleuritic chest pain for 3 days. She was recently discharged 1 month ago following SARS‐CoV‐2 infection with organizing pneumonia requiring high flow oxygen mask and was treated with dexamethasone as per local guidelines. Other past medical history was obesity (BMI 36 kg/m^2^) and diabetes mellitus with latest HbA1c of 8.2%. Her medications were oral hypoglycemic agent (OHA) and insulin therapy.

Upon recent presentation, she was afebrile, tachypneic with respiratory rate 26 breaths/min, tachycardic with heart rate of 105 beats/min and oxygen saturation of 93%–95% on 5 L/min face mask. Clinical examination revealed reduced breath sounds in the right lung. Chest radiograph showed right‐sided hydropneumothorax and a chest drain was inserted which subsequently drained three litres of exudative pleural effusion. Pleural fluid cultures had no growth and were negative for tuberculosis.

**FIGURE 2:**
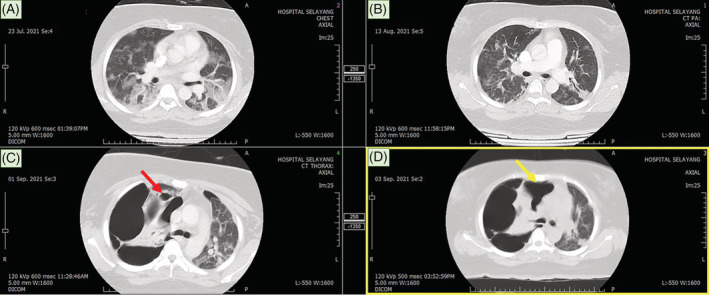
The subsequent CT thorax at day 4 of illness (image A) showed ground‐glass opacity and consolidation with predominant bilateral lower lobe distribution. Radiological improvement noted at day 25 post SARS‐CoV‐2 infection with residual ground‐glass opacity and subpleural curvilinear line (image B). Subsequent CT thorax at day 44 post SARS‐CoV‐2 infection (image C) revealed development of new cystic changes (red arrow) and multiseptated bullae with compressive effect and mediastinal shift. A ruptured cyst was identified leading to right loculated pneumothorax (yellow arrow).

During the hospital stay, serial chest radiographs showed persistent right pneumothorax. CT thorax revealed development of new of cystic changes that rapidly progressed into bilateral lung bullae occupying almost the entire right lung. (Figure 2). Unfortunately, surgical intervention was not suitable in view of bilateral widespread bullous lung disease with increased risk of pneumothorax during single lung ventilation. A CT guided pigtail was inserted for right loculated pneumothorax. She underwent inpatient pulmonary rehabilitation and was discharged after 28 days of hospitalization.

## DISCUSSION

Pneumothorax is a rare complication of SARS‐CoV‐2 infection, which is mainly observed during a severe disease course or in the context of positive pressure ventilation. Chong et al. reported the incidence of pneumothorax to be low (0.3%) but increased up to 12.8%–23.8% in critically ill SARS‐CoV‐2 infected patients requiring invasive mechanical ventilation (IMV).^1^ The mechanisms leading to pneumothorax formation are only partially understood. Diffuse alveolar damage with subsequent air leak has been discussed, and mechanical ventilation causing barotrauma might promote this process.^2^, ^3^ Although alveolar rupture due to barotrauma can occur in the setting of IMV, there are multiple case reports of spontaneous pneumothorax occurring in patients with SARS‐CoV‐2 infection who did not require IMV.^4^, ^5^, ^6^

Others have postulated that the formation of emphysematous bullae or cavitation might be a consequence of pulmonary infarction, which is probably driven by endothelial inflammation.^7^ Comparative pathological findings in lung tissue between SARS‐CoV‐2 and influenza A patients showed similar changes of diffuse alveolar damage with extensive endothelial and vascular damage. These features were absent in uninfected lungs.^8^ Alveolar damage caused by SARS‐CoV‐2 promotes severe destruction of alveolar tissue resulting in bulla formation, thereby enhancing the risk of pneumothorax.^9^

Both our cases were non‐smokers, had no pre‐existing lung disease, and did not receive IMV, therefore ruling out the possibility of mechanical lung injury. Serial CT thorax in our patients showed the progression of lung changes from consolidation to bullous formation and pneumothorax. These concur with recent radiologic studies that have revealed the development of cystic lung changes after SARS‐CoV‐2 infection.^10^ The onset of pneumothorax may vary, either during acute SARS‐CoV‐2 infection or a later presentation ranging from 1 to 6 months.^9^, ^11^, ^12^, ^13^, ^14^, ^15^ It may associate with bullous lung disease or isolated pneumothorax.^16^, ^17^, ^18^, ^19^ Most cases are unilateral, however there are sporadic cases of bilateral pneumothorax.^3^, ^20^

Bullous lung disease can be treated conservatively or surgically. Multiple procedures have been proposed, including local excision of the bullae, plication, stapler resection, lobectomy, and video thoracoscopy.^21^ Surgical therapy is indicated when patients have incapacitating dyspnea or complications related to bullous disease, such as infection or pneumothorax.^22^ Early thoracic surgical opinion should be sought in view of high risk of persistent air leak in complicated pneumothorax cases. However, surgical intervention in newly recovered SARS‐CoV‐2 infected patients poses risk of postoperative complications. A recent international multicenter cohort study of 1128 SARS‐CoV‐2 positive patients undergoing emergent (74%) and elective (26.1%) surgeries noted that pulmonary complications occurred in 51.2% of patients with a 30‐day mortality of 38%, of which 82% of all deaths were due to SARS‐CoV‐2 infection.^23^ Timing of surgery in these cases must be individualized, considering the nature and risk level of the intended surgery, co‐morbidities, and the risk associated with delay of surgery. When possible, surgery should be delayed for at least 7 weeks following SARS‐CoV‐2 infection.^24^

From our case series, the first patient was treated surgically whilst the second patient was treated conservatively. The latter patient was not suitable for surgical intervention in view of bilateral widespread bullous lung disease and increased risk of pneumothorax and hypoxemia during single lung ventilation. Comprehensive pulmonary rehabilitation is feasible with attention to improve exercise performance, lung function and quality of life in patients who develop post SARS‐CoV‐2 pulmonary complications.^25^

In conclusion, the development of bullous lung disease following SARS‐CoV‐2 infection is rare but may be associated with serious morbidity. It should be part of the differential diagnosis in a patient who represents with breathlessness after completing treatment for SARS‐CoV‐2 pneumonia. Patients whose general condition permits should be offered surgical intervention whilst conservative management is reserved for non‐surgical candidates.

## AUTHOR CONTRIBUTION

All listed authors contributed to this article.

## CONFLICT OF INTEREST

None declared.

## ETHICS STATEMENT

The authors declare that appropriate written informed consent was obtained for the publication of this manuscript and accompanying images.

## Data Availability

The data that support the findings of this study are available from the corresponding author upon reasonable request.
